# The association of neutrophils with the development of thyroid nodules: a cross-sectional study

**DOI:** 10.3389/fonc.2026.1849671

**Published:** 2026-08-03

**Authors:** Ling Chen, Xue-xia Gao, Ting-ting He, Jun Su, Ji-bin Huang, Xi Zhang

**Affiliations:** 1 Qiannan Buyi and Miao Autonomous Prefecture Hospital of Traditional Chinese Medicine, Duyun, China; 2 Guizhou University of Traditional Chinese Medicine, Guiyang, China

**Keywords:** alanine aminotransferase, cross-sectional study, neutrophils, serum uric acid, thyroid nodules

## Abstract

**Objectives:**

The prevalence of thyroid nodules (TN) is increasing, necessitating urgent investigation of preventive measures. Evidence has linked the neutrophil-to-lymphocyte ratio to the development of TN. However, the specific role of neutrophils(NEUT) in TN development remains unclear. This study aimed to investigate the association between NEUT levels and TN presence.

**Patients and methods:**

This study included 2,792 participants. Data collected included demographic characteristics (age and gender) and relevant clinical indicators. The association between NEUT levels and TN was analyzed using logistic regression, Restricted Cubic Spline (RCS) models, subgroups and multiplicative interaction analysis.

**Results:**

Univariate logistic regression analysis revealed a significant negative association between NEUT levels and the presence of TN (OR = 0.9149, 95% CI: 0.8598–0.9726). After further adjusting for potential covariates (age, sex, white blood cell count, alanine aminotransferase, serum uric acid, triglycerides, high-density lipoprotein cholesterol, monocyte count, and systolic blood pressure), this inverse association remained statistically significant (OR = 0.8237, 95% CI:0.7116–0.9527). Furthermore, RCS regression analysis indicated a significant linear relationship between NEUT levels and TN risk (*P*-overall = 0.0267). Subgroup and interaction analyses consistently showed an inverse association between NEUT and TN risk across most strata, with significant heterogeneity in the ALT subgroup (I² = 84.6%, *P* = 0.011) but no multiplicative interaction (*P* interaction > 0.05).

**Conclusion:**

Elevated NEUT levels were independently associated with lower odds of TN. This inverse association suggests a potential role of NEUT in the immunological processes related to TN, offering novel insights for further investigation. However, given the cross-sectional design, causality cannot be inferred, and whether modulating NEUT levels may have preventive value remains to be explored in prospective studies.

## 1 Introduction

Thyroid nodules (TN) are among the most common endocrine disorders, with a prevalence exceeding 50% in population-based random sampling studies (1). Clinical data indicate that although most TN are benign, they carry a malignancy risk of approximately 10% (2). Additionally, approximately 5% of cases present with compression symptoms, and another 5% exhibit functional disorders (2). Moreover, existing research indicates that the incidence and mortality rates of thyroid cancer have been steadily increasing (3, 4). Without effective interventions, the rising prevalence of TN is emerging as a significant public health challenge and impose a substantial economic burden (3). It is therefore essential to identify effective preventive measures for TN. Neutrophils(NEUT) are the most abundant immune cell in human peripheral blood, accounting for approximately 50-70% of the total circulating white blood cell population (5, 6). As a core component of the innate immune system, NEUT eliminate pathogens *via* multiple effector pathways (5). Furthermore, NEUT participate in regulating various pathophysiological processes, including acute injury repair, tumorigenesis, autoimmune diseases, and chronic inflammation (7). Recent research suggests that the neutrophil-to-lymphocyte ratio (NLR) is associated with TN development (8). However, evidence regarding the association between NEUT and TN development is limited. We conducted a cross-sectional study to explore the potential association between NEUT levels and TN risk.

## 2 Patients and methods

### 2.1 Study participants

The data for this study were collected from individuals who underwent health examinations at the Qiannan Buyi and Miao Autonomous Prefecture Hospital of Traditional Chinese Medicine between January 1, 2022, and December 31, 2022. The inclusion criteria were: (1) Underwent thyroid color Doppler ultrasound; (2) Completed comprehensive laboratory testing, including complete blood cell counts and full metabolic function assessments. The exclusion criteria were: (1) Missing key data; (2) Clinically implausible laboratory values. After screening, 2,792 individuals were included in the study, among whom 1,851 (66.29%) were diagnosed with TN *via* thyroid color Doppler ultrasound.

### 2.2 Clinical measurements and diagnostic criteria

Blood pressure was measured with an OMRON HBP-9020 fully automatic electronic sphygmomanometer after participants rested quietly for 5 minutes. Venous blood samples were collected after an overnight fast of at least 8 hours. Subsequently, venous blood samples were analyzed for a complete blood count using a Sysmex XN-3000 hematology analyzer. Biochemical tests were performed on a Roche Cobas 702 fully automated biochemical analyzer. Additionally, thyroid ultrasound was conducted with an Apogee 3800 color Doppler ultrasound system.

We collected demographic characteristics (age, gender) and relevant clinical indicators from the subjects, including body mass index (BMI), white blood cell count (WBC), alanine aminotransferase (ALT), aspartate aminotransferase (AST), serum uric acid (SUA), creatinine (Cr), triglycerides (TG), total cholesterol (TC), low-density lipoprotein cholesterol (LDL-C), high-density lipoprotein cholesterol (HDL-C), total bilirubin (TBIL), monocytes (MONO), systolic blood pressure (SBP), diastolic blood pressure (DBP), lymphocytes (LYMPH), and NEUT. Thyroid ultrasound is the preferred initial imaging modality for thyroid disorders in clinical practice, and is therefore widely used for the detection and characterization of nodular diseases (9, 10). TN are localized lesions within the thyroid gland that exhibit clear imaging demarcation from the surrounding thyroid parenchyma (11).

### 2.3 Statistical analysis

For baseline characteristics, categorical variables were presented as n (%) and compared using the chi-square test. Continuous variables with non-normal distributions were described as median (interquartile range [IQR]) and compared between groups using the Mann-Whitney U test. Univariate and multivariate logistic regression models were used to assess the association between NEUT counts (independent variable) and TN presence (dependent variable). All statistical analyses, including chi-square tests, Mann-Whitney tests, and logistic regression, were performed with GraphPad Prism software (version 10.1.2). To investigate the potential nonlinear association between NEUT counts and TN, we used logistic regression with restricted cubic splines (RCS) in R (version 4.4.3) to model the dose-response relationship. We adjusted for confounding factors in the construction of both the logistic regression and RCS models, with statistical significance defined as *P* < 0.05. Subgroup analysis and multiplicative interaction analysis were used to examine the relationship between NEUT and the risk of TN across the following subgroups: gender, age (≤60 years or >60 years), WBC (≤6.5 10^^9^/L or>6.5 10^^9^/L), ALT (≤20.2 U/L or>20.2 U/L), SUA(≤362 umol/L or>362 umol/L), TG (≤1.66 mmol/L or>1.66 mmol/L), HDL-C (≤1.23 mmol/L or>1.23 mmol/L), MONO (≤0.46 10^^9^/L or>0.46 10^^9^/L), SBP (≤124 mmHg or>124 mmHg). The I² statistic and Q test were employed to assess heterogeneity among subgroups. Notable heterogeneity was considered to exist if the *p* value for the Q statistic was < 0.05 or the I² value exceeded 50%. Multiplicative interaction analysis was conducted based on the identified heterogeneity. Subgroup analysis and multiplicative interaction analysis were performed using R (version 4.4.3). The threshold for statistical significance was set at *p* < 0.05.

## 3 Results

### 3.1 Baseline characteristics

**Table 1** summarizes the demographic characteristics and clinical baseline data of the subjects in this study. Of the 2,792 participants, they were classified into the thyroid nodule (TN) group (n=1,212, 43.4%) and non-TN group based on thyroid color Doppler ultrasound findings (n=1,580, 56.6%). Statistical analysis showed that participants with TN were significantly older than those without TN (*p* < 0.05). Moreover, the TN group had a significantly higher proportion of females than the non-TN group (*p* < 0.05). Several key clinical laboratory parameters, including WBC, ALT, AST, SUA, CR, TG, HDL-C, MONO, SBP, and NEUT, differed significantly between the two groups (*p* < 0.05).

**Table 1:** Baseline characteristics of the study subjects.

Variables	Overall (n = 2792)	Non-TN (n=1580)	TN (n = 1212)	*P*
Age (years), M (IQR)	46.00 (34.00, 57.00)	41.00 (32.00, 53.00)	52.00 (38.00, 61.00)	*<0.0001*
Male, n (%)	1851 (66.30)	1163 (73.61)	688 (56.77)	*<0.0001*
BMI (kg/m^2^), M (IQR)	24.46 (22.31, 26.72)	24.56 (22.27, 26.76)	24.39 (22.35, 26.68)	0.6569
WBC (10^9/L),M (IQR)	6.50 (5.54, 7.58)	6.59 (5.60, 7.67)	6.39 (5.46, 7.45)	*0.0009*
ALT (U/L),M (IQR)	20.20 (13.78, 31.20)	21.30 (14.20, 33.51)	18.90 (13.50, 28.90)	*<0.0001*
AST (U/L),M (IQR)	21.95 (18.10, 27.20)	22.20 (18.30, 27.63)	21.70 (17.90, 26.50)	*0.0181*
SUA (umol/L),M (IQR)	362.00 (298.00, 426.00)	371.00 (304.00, 431.99)	354.00 (290.00, 417.09)	*<0.0001*
CR (umol/L), M (IQR)	81.00 (68.00, 93.00)	82.94 (70.00, 93.00)	79.00 (66.00, 92.00)	*<0.0001*
TG (mmol/L),M (IQR)	1.66 (1.08, 2.52)	1.71 (1.10, 2.60)	1.58 (1.06, 2.40)	*0.0075*
TC (mmol/L),M (IQR)	4.84 (4.20, 5.47)	4.79 (4.18, 5.46)	4.88 (4.23, 5.49)	0.2858
LDL-C (mmol/L),M (IQR)	2.87 (2.35, 3.41)	2.85 (2.34, 3.39)	2.90 (2.35, 3.43)	0.2906
HDL-C (mmol/L),M (IQR)	1.23 (1.05, 1.45)	1.21 (1.03, 1.43)	1.25 (1.06, 1.48)	*0.0022*
TBIL (umol/L),M (IQR)	10.70 (8.20, 14.10)	10.80 (8.30, 14.40)	10.65 (8.20, 13.80)	0.1389
MONO (10^9/L),M (IQR)	0.46 (0.38, 0.57)	0.47 (0.39, 0.57)	0.45 (0.37, 0.55)	*0.0007*
SBP (mmHg),M (IQR)	124.00 (113.00, 137.00)	122.00 (112.00, 135.00)	127.00 (114.75, 141.00)	*<0.0001*
DBP (mmHg),M (IQR)	76.00 (68.00, 85.00)	75.00 (68.00, 84.00)	76.00 (69.00, 85.00)	0.0532
LYMPH (10^9/L),M (IQR)	2.12 (1.74, 2.58)	2.13 (1.76, 2.60)	2.09 (1.73, 2.56)	0.0566
NEUT (10^9/L),M (IQR)	3.61 (2.91, 4.39)	3.68 (2.95, 4.45)	3.52 (2.87, 4.29)	*0.0019*

TN, Thyroid Nodules; BMI, Body Mass Index; WBC, White Blood Cell Count; ALT, Alanine Aminotransferase; AST, Aspartate Aminotransferase; SUA, Serum Uric Acid; CR, Creatinine; TG, Triglycerides; TC, Total Cholesterol; LDL-C, Low-Density Lipoprotein Cholesterol; HDL-C, High-Density Lipoprotein Cholesterol; TBIL, Total Bilirubin; MONO, Monocytes; SBP, Systolic Blood Pressure; DBP, Diastolic Blood Pressure; LYMPH, Lymphocytes; NEUT, Neutrophils. Italicized values represent statistically significant differences, with P < 0.05.

### 3.2 Association of NEUT with TN

**Figure 1:**
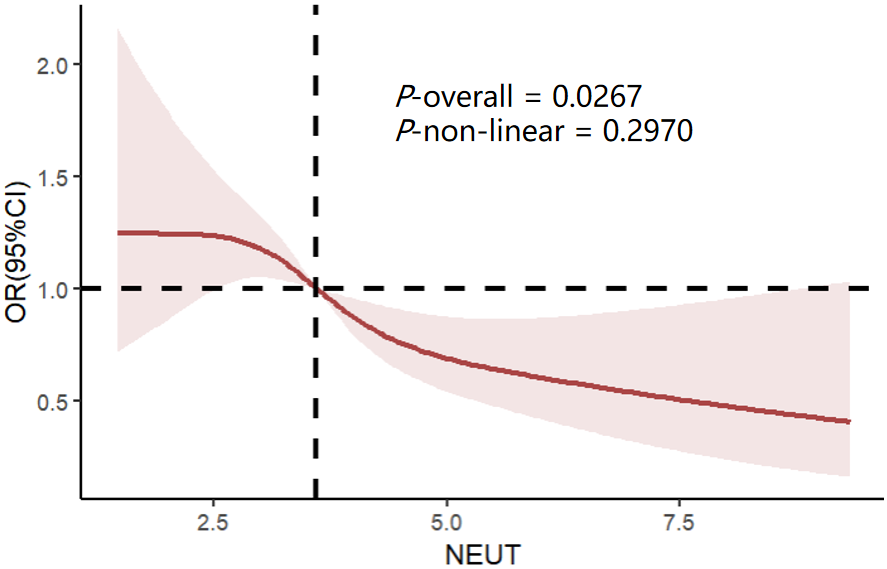
Restricted cubic spline plot between NEUT and TN. TN, Thyroid Nodules; NEUT, Neutrophils.

Logistic regression was used to investigate the relationship between NEUT levels and TN. As shown in **Table 2**, univariate logistic analysis revealed a negative association between NEUT levels and TN presence (OR: 0.9149, 95%CI: 0.8598–0.9726). After adjusting for age, sex, WBC, ALT, SUA, TG, HDL-C, MONO, and SBP in the multivariate logistic regression model, NEUT counts remained significantly associated with TN presence (OR = 0.8237, 95% CI: 0.712–0.953). Other factors independently associated with TN in the multivariate model included older age (OR 1.033, 95% CI 1.027–1.040), female sex (OR 0.4111, 95% CI 0.3339–0.5052), and higher systolic blood pressure (SBP) (OR 1.006, 95% CI 1.001–1.011) (**Table 2**). RCS regression model was applied to quantitatively analyze the potential nonlinear dose-response relationship between NEUT counts and TN risk. After adjustment for confounders, a significant linear dose-response relationship was observed between NEUT counts and TN risk (*P*-overall = 0.0267) (**Figure 1**).

**Table 2:** Univariate and multivariate logistic analysis of TN.

Variables	Univariate analysis		Multivariate analysis	
	OR (95% CI)	*P* value	OR (95% CI)	*P* value
Age	1.035 (1.029 - 1.040)	*<0.0001*	1.033 (1.027 - 1.040)	*<0.0001*
Male Gender	0.4708 (0.4012 - 0.5520)	*<0.0001*	0.4111 (0.3339 - 0.5052)	*<0.0001*
BMI	0.9983 (0.9763 - 1.021)	0.8793		
WBC	0.9268 (0.8846- 0.9705)	*0.0013*	1.133 (0.9980 - 1.286)	0.0538
ALT	0.9930 (0.9889 - 0.9970)	*0.0007*	1.001 (0.9962 - 1.005)	0.7896
AST	0.9934 (0.9859 - 1.001)	0.0864		
SUA	0.9982 (0.9974 - 0.9990)	*<0.0001*	1.000 (0.9994 - 1.001)	0.4539
CR	0.9978 (0.9939 - 1.001)	0.2123		
TG	0.9550 (0.9175 - 0.9915)	*0.0196*	0.9661 (0.9222 - 1.009)	0.1307
TC	1.011 (0.9381 - 1.090)	0.7680		
LDL-C	1.025-(0.9365 - 1.122)	0.5893		
HDL-C	1.449 (1.151 - 1.827)	*0.0016*	0.8639 (0.6454 - 1.155)	0.3245
TBIL	0.9862 (0.9722 - 1.000)	0.0552		
MONO	0.4073 (0.2445 - 0.6749)	*0.0005*	0.6008 (0.2807 - 1.281)	0.1879
SBP	1.013 (1.009 - 1.017)	*<0.0001*	1.006 (1.001 - 1.011)	*0.0123*
DBP	1.003 (0.9993 - 1.008)	0.1573		
LYMPH	0.9029 (0.8036 - 1.014)	0.0845		
NEUT	0.9149 (0.8598 - 0.9726)	*0.0046*	0.8237 (0.7116 - 0.9527)	*0.0091*

TN, Thyroid Nodules; BMI, Body Mass Index; WBC, White Blood Cell Count; ALT, Alanine Aminotransferase; AST, Aspartate Aminotransferase; SUA, Serum Uric Acid; CR, Creatinine; TG, Triglycerides; TC, Total Cholesterol; LDL-C, Low-Density Lipoprotein Cholesterol; HDL-C, High-Density Lipoprotein Cholesterol; TBIL, Total Bilirubin; MONO, Monocytes; SBP, Systolic Blood Pressure; DBP, Diastolic Blood Pressure; LYMPH, Lymphocytes; NEUT, Neutrophils. Italicized values represent statistically significant differences, with P < 0.05.

### 3.3 Subgroup analysis

**Figure 2:**
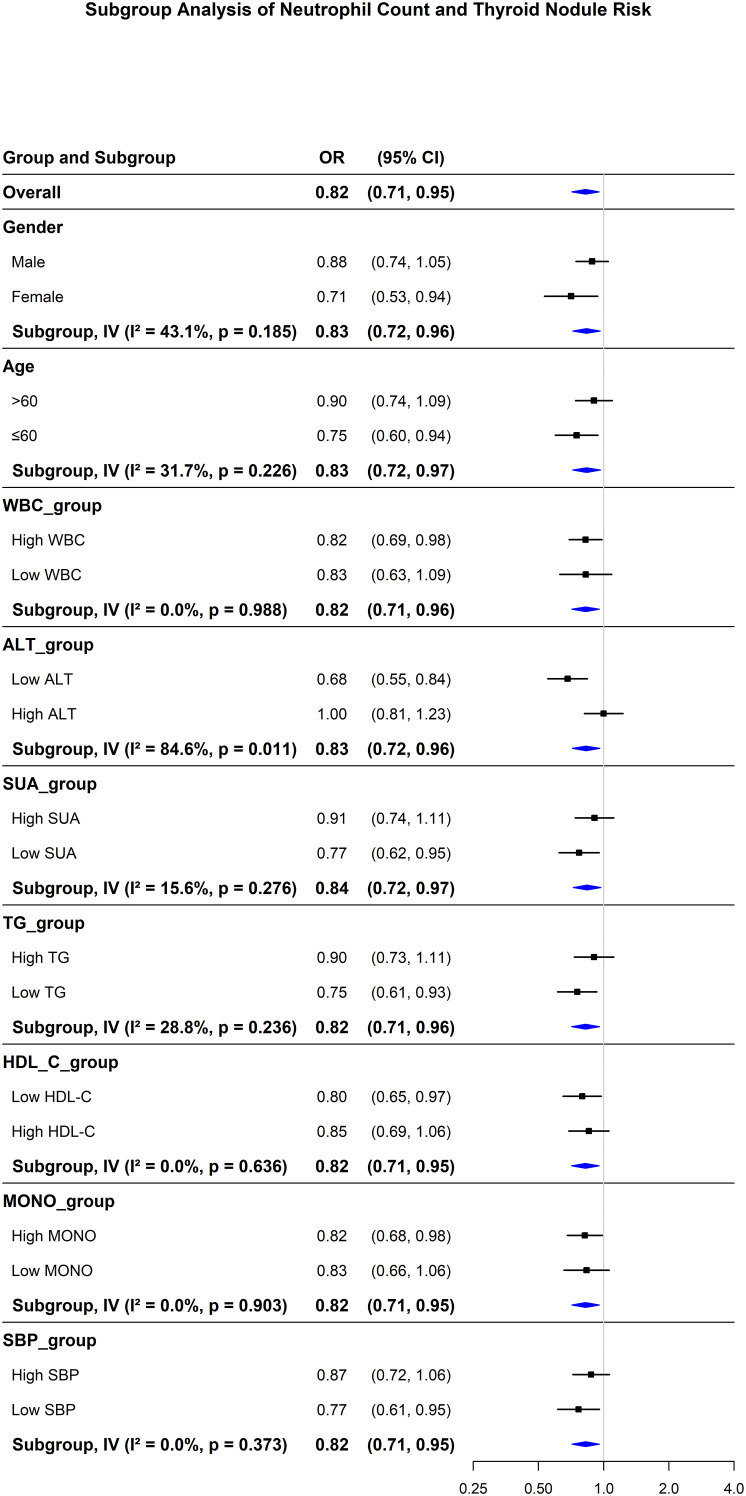
Subgroup analysis of neutrophil count and thyroid nodule risk. WBC, White Blood Cell Count; ALT, Alanine Aminotransferase; SUA, Serum Uric Acid; TG, Triglycerides; HDL-C, High-Density Lipoprotein Cholesterol; MONO, Monocytes; SBP, Systolic Blood Pressure.

Subgroup analysis was performed to verify the robustness of the relationship between NEUT and TN risk. The inverse association between NEUT and TN risk was consistent across strata of gender, age, WBC, ALT, SUA, TG, HDL-C, MONO and SBP. Significant inverse associations were observed not only in females, but also in subjects with age ≤ 60, ALT ≤ 20.2 U/L, SUA ≤ 362 μmol/L, TG ≤ 1.66 mmol/L or SBP ≤ 124 mmHg. Adjusted *ORs* were estimated to be 0.71, 0.75, 0.68, 0.77, 0.75 and 0.77, respectively (**Figure 2**).

### 3.4 Interaction analysis

Based on subgroup analysis, significant heterogeneity was detected in the ALT subgroup (I² = 84.6%, P = 0.011), suggesting a potential interaction between ALT and NEUT in relation to TN risk. Further multiplicative analysis was performed to validate the robustness of the association between NEUT and TN risk. The results showed multiplicative interactions were not observed (P for interaction > 0.05) (**Table 3**).

**Table 3:** Multiplicative interaction between ALT and NEUT.

Variables		TN n (%)	Non-TN n (%)	OR (95% CI)	*P*_interaction_
ALT	NEUT				
Low	Low	369 (50.0)	369 (50.0)	1.00	
High	Low	279 (41.8)	388 (58.2)	0.72 (0.58-0.89)	0.0022
High	High	272 (37.7)	449 (62.3)	0.61 (0.49-0.75)	<0.001
Low	High	292 (43.8)	374 (56.2)	0.78 (0.63-0.96)	0.0211
ALT×NEUT				1.08 (0.80-1.46)	0.620

ALT, alanine aminotransferase; NEUT, neutrophils; TN, thyroid nodules.

## 4 Discussion

This cross-sectional study examined the investigated the association between NEUT counts and TN presence. Our results showed that NEUT counts were independently and inversely associated with TN risk, and this association persisted after adjustment for multiple confounders. Furthermore, RCS analysis identified a significant linear dose-response relationship between NEUT counts and the risk of TN. This finding differs from a previous study, which reported no significant association between the neutrophil-to-lymphocyte ratio (NLR) and TN presence (8). The discrepancy may be attributed to the different indicators assessed: our study focused on absolute NEUT counts, whereas the prior study examined NLR (a composite index of NEUT and lymphocytes), which may reflect distinct inflammatory profiles.

Subgroup analysis revealed that NEUT were inversely associated with the risk of TN, and this inverse association remained consistent across most subgroups, with substantial heterogeneity observed in the ALT subgroup. Specifically, in individuals with normal ALT levels, NEUT was inversely associated with TN (OR = 0.68, 95% CI: 0.55–0.84); in contrast, this association was absent in those with elevated ALT levels (OR = 1.00, 95% CI: 0.81–1.23). NEUT is the most abundant immune cell type in the human circulation and exerts diverse biological functions (12). We hypothesize that systemic metabolic disorders or inflammatory states, as indicated by abnormal liver function (marked by elevated ALT), may alter the functional role of NEUT in the TN microenvironment.

Within the subgroup analysis, we further demonstrated that the inverse association between NEUT and TN risk was stronger in females (OR = 0.71) than in males (OR = 0.88), which implies a potential sexual dimorphism in this protective effect. Accumulating evidence has shown that functional NEUT subsets are tightly regulated by gender; for example, in Alzheimer’s disease, a specific IL-17^+^ NEUT subset preferentially drives pathological progression in females (13); whereas in bladder cancer, a male-enriched senescent-like NEUT subset mediates a more immunosuppressive microenvironment, and this gender difference is regulated by the gut microbiota-metabolite axis (14). Thus, we hypothesize that the pronounced protective effect observed in females in the present study may result from the preferential activation of a specific NEUT subset regulated by sex hormones or the gut microbiome, which thereby more effectively inhibits TN development.

As vital components of the immune system, NEUT play crucial roles in inflammatory responses and maintaining homeostasis (15). They act as the first responders to inflammation (16). *In vitro* studies have demonstrated that neutrophil-derived exosomes/extracellular vesicles exhibit immunomodulatory properties, exerting dual pro-inflammatory and anti-inflammatory regulatory effects on both the monocyte-macrophage system and NEUT themselves (17, 18). Notably, tumor necrosis factor (TNF)-induced necroptosis in NEUT can suppress systemic inflammatory responses by shortening the lifespan of NEUT and reducing proinflammatory cytokine production (19, 20). Given that systemic inflammation is known to contribute to TN development (21). we hypothesize that NEUT (elevated NEUT counts) may reduce TN risk by attenuating systemic inflammatory responses.

Despite identifying a significant inverse association between NEUT levels and TN risk after rigorous adjustment for confounders, the following limitations warrant cautious interpretation of our results: (1) This study was conducted at a single center, which may limit the generalizability of our findings to more diverse populations. (2) Few studies have specifically investigated the association between NEUT counts and TN, which hinders direct comparison of our results with previous literature. (3) The cross-sectional design only allows us to demonstrate an association between NEUT counts and TN risk, but precludes the establishment of a causal relationship. Notwithstanding these limitations, our study indicates that elevated NEUT counts are independently associated with lower odds of TN, a finding that may inform future etiological and preventive research. (4) Residual confounding may persist, as data on confounders including smoking, alcohol consumption, thyroid function, thyroid autoantibodies, iodine intake, diabetes, medications affecting leukocyte counts, acute or chronic inflammatory diseases, and infection status were not collected in this preliminary survey. However, this conclusion warrants validation in large-scale, multi-center prospective cohort studies.

## Data Availability

The raw data supporting the conclusions of this article will be made available by the authors, without undue reservation.
